# A Numerical Analysis of Resin Flow in Woven Fabrics: Effect of Local Tow Curvature on Dual-Scale Permeability

**DOI:** 10.3390/ma14020405

**Published:** 2021-01-15

**Authors:** Hatim Alotaibi, Masoud Jabbari, Constantinos Soutis

**Affiliations:** 1 Department of Mechanical, Aerospace and Civil Engineering, The University of Manchester, Manchester M13 9PL, UK; hatim.alotaibi@postgrad.manchester.ac.uk; 2 Department of Materials, The University of Manchester, Manchester M13 9PL, UK; constantinos.soutis@manchester.ac.uk; 3 University of Manchester Aerospace Research Institute, The University of Manchester, M13 9PL Manchester, UK

**Keywords:** permeability, dual-scale porous media, porosity, multi-scale numerical modelling, resin transfer moulding, tow undulation

## Abstract

Permeability is a crucial flow parameter in liquid composite moulding (LCM), which is required to predict fibre impregnation, void formation and resin back flow. This work investigates the dual-scale (micro- and meso-) nature of permeability during resin infusion into woven fabric by incorporating the intra tow flow where the degree of local tow curvature (tow/yarn undulation) is taken into account. The mesoscopic permeability of a dual-scale porous media in a unit cell is estimated using Darcy’s law, where the Gebart analytical model is applied for the intra tow flow in longitudinal and transverse directions with respect to distinct fibre packing arrangements. The results suggest that for a low fibre volume fraction (≤42%), the degree of local curvature at the mesoscale can be neglected. However, for a high fibre volume fraction (>42%) and a higher fibre bundle curvature, the proposed model should be adopted, since the resin flow is affected by a mesoscopic tow curvature that could result in around 14% error in predicting permeability. It is shown that the permeability results of the current study are in good agreement with and in the range of the retrieved available experimental data from the literature.

## 1. Introduction

### 1.1. Background

The formation of voids and dry spots during liquid composite moulding (LCM) should be avoided in the effort to acquire a defect-free composite part. Aiming for a high-performance composite, one must consider the fibrous porous media in LCM and the so-called dual-scale flow in porous media [1,2,3]. The appearance of dry zones during the mould filling process are caused by potential factors such as injection port location inlet/outlet, preform permeability, resin flow rate, and resin viscosity [4,5]. This filling process throughout the mould is described as a flow impregnation process that is categorised into two flow regions (dual-scale flow): one is the inter-yarn/tow referring to a (free-) flow between the yarns/tows spacing (meso-scale) with a relatively high porosity. Whereas, intra- yarn/tow corresponds to a flow between the filaments/fibres (micro-scale) that is considered as a low-porosity region.

### 1.2. Dual-Scale Porous Media Modelling

**Figure 1:**
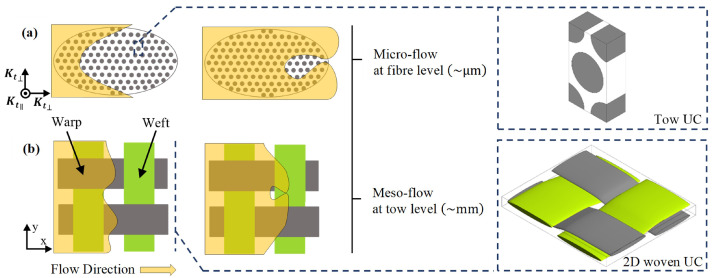
Void formation (**a**) inside the intra-tow region (microscopic level), (**b**) between the yarns (mesoscopic level).

While Darcy’s law has been successfully applicable for single-scale flow modelling in LCM [6,7,8,9], in dual-scale flow modelling, the applicability is obtained by applying an additional source term to Navier–Stokes (N–S) equations in order to model flow at tow and fibre levels. This dual-scale flow nature (micro- and meso-scale) is the core approach to be implemented in numerical modelling when simulating resin impregnation in composite preforms [2,10,11]. The unit cell or the so-called representative volume element (RVE) is defined as the smallest volume at fibre/tow level in which a measurement can be taken to represent the entire volume (macro-scale). Figure 1 graphically illustrates that the distance between fibre bundles is in the order of millimetres, but in micrometres between fibres (within the yarn), causing slow intra-tow impregnation, such a delay of impregnation within the intra-tow region results in void formation and partial saturation beyond the flow front [10,12,13].

Parnas and Phelan [10] proposed an approach for calculating flow that enters a fibre bundle or a tow by using a sink term, this term is defined for advancing flows that are drained or leaked by fibre bundles that acted as sinks; they concluded that the behaviour of the flow is radial across the tows. Pillai and Advani [14] applied the sink term for a two-layer model based on a rectangular cross-section that was applied to other cross sections. The two-layer model adopts the dual-scale porous media, thus a small drop in the pressure inlet profile in resin transfer moulding (RTM) was observed. Wang and Grove [15] defined transient tow impregnation as a function of the saturation rate in a 2-D unit cell, and the saturation rate was incorporated into continuity equation for the connectivity of macro-micro pores as a sink term. It has been found that models which assume solid tows underestimate the driven influence by the micro-flow (intra-tow) on the meso-flow (inter-tow), and particularly at high fibre volume fractions within the fibre bundles.

Researchers have demonstrated either experimentally or numerically that the pressure–time relationship is linear in single-scale flow problems (non-woven unidirectional composites), but becomes non-linear in a dual-scale porous medium, such as woven fabrics or stitched ones [4,16]. Unidirectional or radial flow experiments for woven fabrics showed a nonlinear relationship, as reported by Parseval et al. [13], Parnas et al. [17], and Zhong et al. [18], where the nonlinearity in the pressure profile is caused by unsaturated tows. In experiments of random fibre mats, linearity is obtained by Parseval et al. [13] and Wu et al. [19], where for the analysis, an isotropic porous media is assumed as well as constant flow rate. It is summarised that Darcy’s law combined with the continuity equation unsuccessfully estimates the pressure profile (and hence flow impregnation) in dual-scale that necessitates adding an additional source term (sink term) to the system of equations (continuity and momentum equations).

It appears from these studies that flow front progression or saturation rate is a key element that affects resin flow impregnation and may explain fabric deformation and void formation. Spaid and Phelan [20] thought that a combination of pressure gradient, capillary effects and degree of saturation are important resin flow parameters during manufacturing. Their study presented a novel technique to characterise flows in heterogeneous porous media by employing the developed Stokes/Brinkman equation via the Lattice Boltzmann method. However, the accurate prediction of inhomogeneous saturation is relatively hard, which leads researchers to a wrong assumption of homogenous resin flow throughout the porous media [21].

Global permeability is affected by the pressure nonlinearity that is prompted by the dual-scale (micro/meso) approach. Dual-scale flow (permeable tows) shows a considerable difference in comparison to single-scale flow (impermeable tows), which ranges from 3% to 30% [22,23,24]. Thus, the intra-yarn flow must be taken into account together with inter-yarn flow when calculating the overall permeability in a porous medium [10,14,25].

Previous studies [26,27,28,29,30,31] investigated the rotational angle that appears in the anisotropic macro-flow via channel or radial injections within the RTM process, in which the in-plane permeability acts differently and is affected by or dependent on the degree of anisotropy and angle of rotation, and, therefore, this can take place in micro-level during resin impregnation. As the nature of woven fabrics exhibits curvatures (waviness) within the yarns by virtue of either thickening or binding the fibre bundles, this paper is motivated to discuss the significant impact of the local flow on the global one, wherein the degree of curvature is incorporated at the microscopic level. As said, intra-tow permeability is a crucial factor that can affect the macroscopic resin flow front and hence the calculated overall permeability.

Table 1 describes the influence of intra-tow flow on the overall permeability based on different fibre packing arrangements and numerical models. $K_{o}$ indicates the permeability considering both inter- and intra-tow flow, $K_{s}$ is the single-scale permeability assuming inter-tow flow with solid (impermeable) fibre bundles, $\phi_{o}$ represents the overall porosity of the medium (intra- and inter-yarn empty regions) in a unit cell, and $\phi_{t}$ is the intra-tow porosity (intra-yarn region) to be filled by resin.

## 2. Methodology

### 2.1. Continuity and Momentum Equations

In RTM, resin flow behaves in a laminar manner, since the fluid viscous forces are dominating the inertial forces. Thus, in the impregnation process, a low Reynold number is seen, (Re≪1), in which the fluid is categorised as creeping or viscous flow. In general, the continuity and momentum equations for laminar flow are presented below

(1) $$\frac{\partial }{\partial t}\left(\rho u\right)+\nabla \cdot \left(\rho uu\right)=-\nabla p+\nabla \cdot \tau +\rho g+S$$

(2) $$\frac{\partial \rho }{\partial t}+\nabla \cdot \left(\rho u\right)=0$$

where τ is the stress tensor, ρg is the body force term, $\nabla \cdot \left(\rho uu\right)$ is the convective acceleration term, $\frac{\partial }{\partial t}\left(\rho u\right)$ is the local acceleration, ∇p is the pressure gradient, and *S* is the source term. For steady-state, incompressible, creeping flow with Newtonian behaviour and ignoring the body force term, the continuity and momentum equations are reduced as follows:

(3) $$0=-\nabla p+\mu \nabla^{2}u+S$$

(4) ∇·u=0

Equation (3) is the so-called Stokes equation and can be used to simulate inter-yarn flow where there is no porous region (and term *S* is ignored). The additional momentum source term *S* is provided to model porous media, which normally consists of the viscous loss term, shown below

(5) $$S=-\frac{\mu }{K_{t}}u$$

where $\frac{1}{K_{t}}$ is the viscous resistance, and u is the volume averaged velocity. Inserting Equation (5) into Equation (3) leads to the so-called Darcy–Brinkman equation for porous media, which is the generalisation of Darcy’s law to facilitate the matching boundary conditions between the larger pores (inter-yarn region) and the permeable medium (intra-yarn region):

(6) $$\mu \nabla^{2}u-\frac{\mu }{K_{t}}u=\nabla p$$

in which $K_{t}$ is the permeability tensor of the tow. The tow permeability is generally estimated using analytical, semi-analytical and empirical solutions of the ideal tow topologies [35,36,37]. Thus, without inertial terms—which are generally employed when free-flow regions are not included—we get the renowned Darcy’s law [38,39,40]

(7) $$u=-\frac{K_{o}}{\mu }\nabla p$$

where u the volume-averaged velocity vector, ∇p is the pressure gradient, μ is the viscosity of the fluid, and $K_{o}$ is the global permeability tensor. Permeability tensor could be either symmetric or orthotropic; however, the latter seems to be more realistic due to imperfectly layered porous media [41]. Neglecting all off-diagonal components of the permeability tensor ($K_{ij}$), Equation (7) becomes the following:

(8) $$\left[\begin{array}{c} u_{x}\\ u_{y}\\ u_{z} \end{array}\right]=-\frac{1}{\mu }\left[\begin{array}{ccc} K_{xx} & 0 & 0\\ 0 & K_{yy} & 0\\ 0 & 0 & K_{zz} \end{array}\right]\times \left[\begin{array}{c} \partial p/\partial x\\ \partial p/\partial y\\ \partial p/\partial z \end{array}\right]$$

where $K_{xx}$ and $K_{yy}$ are in-plane permeabilities and $K_{zz}$ is the through thickness or transverse permeability.

### 2.2. Permeable and Impermeable Tows

The numerical modelling presented in this paper is performed in ANSYS-Fluent 19.2, in which a momentum source term is added to Naiver–Stokes equations to enable modelling of porous regions. Viscous resistance—second term in the left-hand side of Equation (6)—is the required input for the porous model, and that is generally determined by any of the theoretical models for calculating the microscopic permeability (flow intra-tow), whereby the viscous resistance is equal to the inverse of flow intra-tow ($1/K_{t}$). In this manner, the flow can be modelled inside and in-between the tows, allowing the prediction of the overall permeability $K_{o}$ in the dual-scale porous media using the Darcy–Brinkman equation.

The porous regions are denoted by $\phi_{s}$, inter-tow porosity, $\phi_{t}$, intra-tow porosity, and $\phi_{o}$, the overall/aggregate porosity. The overall porosity of the dual-scale medium is calculated as shown below in Equation (9) [24]. Since this study adopted the assumptions of a homogeneous isotropic porous medium and a single-phase fluid flow problem (perfect fibre-matrix bonding), the overall porosity ($\phi_{o}$) relationship to fibre volume fraction becomes $V_{f}=1-\phi_{o}$.

(9) $$\phi_{o}=\phi_{s}+\phi_{t}-\phi_{s}\phi_{t}$$

It should be noted that variation of intra-tow porosities would not numerically affect the geometrical details, but could influence the fibre volume fraction. The intra-tow porosity contributes to the overall/aggregate porosity—Equation (9)—hence the fibre volume fraction. As explained in Section 2.1, yarns within the unit cell are set as porous zones, in which the porous model allows varying or specifying intra-tow porosity and permeability values. Unit cell dimensions used in Section 3.2 and Section 3.3 kept the same, whereas the cross-sectional dimensions of yarns (warp/weft) vary to account for different inter-tow porosities (e.g., 66%, and 30%).

**Figure 2:**
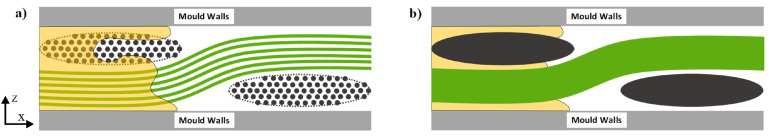
Flow regions for permeability prediction: (**a**) permeable tows, (**b**) impermeable tows ($\phi_{t}=0$).

To assure the robustness and applicability of the developed numerical tool, a numerical analysis was carried out for a more complex fabric geometry to demonstrate its employability in any other complex fabric architectures. A unit cell of plain or 2D woven fabric is modelled with regard to impermeable and permeable tows (c.f. Figure 2) to investigate the effect of the microscopic permeability (intra-tow flow) on the mesoscopic permeability (inter-tow flow).

The work by Belov et al. [23] is selected for the purpose of comparing and validating the developed numerical tool and approach that are presented in this study. The authors studied the permeability in a unit cell of a plain-woven fabric for a range of fibre volume fractions as well as tow permeability values. Thus, three longitudinal and transverse microscopic permeability values are given for each unit cell fibre volume fraction, ranging from solid ($K_{t}=0$) to highly permeable tows to analyse the influence of local permeability on the global permeability. In the study by Belov et al. [23], the lattice Boltzmann method (LBM) was used, and woven fabric dimensions, material and flow type, and density and viscosity were all obtained from RTM experimental studies by Hoes et al. [42] and Luo et al. [43].

Therefore, the case studies for woven fabrics in the present work are based on the same assumptions at the perspective of flow and woven fabric properties, but in a different implementation in which the commercial software package ANSYS is adopted. Moreover, this study offers greater customization of the numerical model, in which resin impregnation within the woven fibre bundles is tracked and assigned as a function of an angle (θ), which is needed in event of curvature or tangent.

### 2.3. Local Curvature

**Figure 3:**
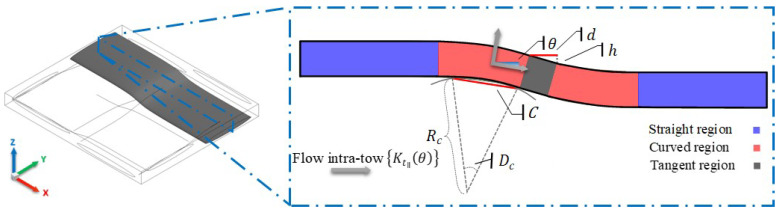
Schematic diagram for the intra-tow flow illustrating yarn curvature.

To account for the local curvature (undulation) of the tows, a user-defined function (UDF) code is implemented via the C computer programming language for the porous woven tows. The UDF incorporates the intra-tow flow inside the woven fabric architecture Figure 3, where the degree of local curvature of tows can affect the flow. In a separate section, a comparative study is conducted for the structures with a range of low to high fibre volume fractions ($0.55\leq V_{f}\leq 0.9$) to investigate their influence on the resin flow, which contributes to the manufacturing quality and performance of the designed component.

**Figure 4:**
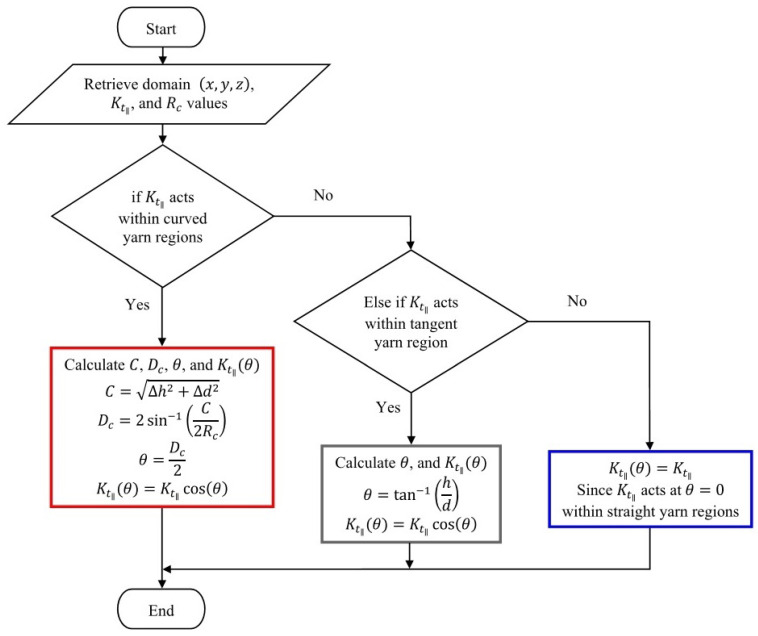
User-Defined Function (UDF) flow chart.

Intra-tow porosity $\phi_{t}$ is selected from low to high porous tows to investigate the influence on flows along fibres using, initially, the default porous model by ANSYS-Fluent in which Gebart’s model [35] is employed in viscous resistance formulation, $1/K_{t}$, for parallel, $K_{t\|}$, and perpendicular, $K_{t⊥}$, intra-tow permeabilities. The same manner is adopted for the UDF case with exception to the parallel $K_{t\|}$ intra-tow permeability as it is controlled by an algorithmic code based on degree of curvature and relevant equations. In straight tows, $K_{t\|}$ is a function of $R_{f}$ and $\phi_{t}$, but $K_{t\|}$ becomes a function of $R_{f}$, $\phi_{t}$ and θ in curved and inclined tows. Wherein $K_{t⊥}$, flow is acting almost the same in both straight and curved tows, as it is propagating perpendicularly to fibres and that would show identical permeabilities.

(10) $$\left\{\begin{array}{ccccc} D_{c}=2\sin^{-1}\left(\frac{C}{2R_{c}}\right), & \theta =\frac{D_{c}}{2} & \; & & \left(a\right)\\ \theta =\tan^{-1}\left(\frac{h}{d}\right) & \; & & \; & \left(b\right)\\ K_{t\|}\left(\theta \right)=K_{t\|}\cos\left(\theta \right) & \; & & \; & \left(c\right)\\ K_{t⊥}\left(\theta \right)=K_{t⊥} & \; & & \; & \left(d\right) \end{array}\right.$$

where $D_{c}$ is the degree of curvature, *C* is the chord length, $R_{c}$ is the radius of curvature, θ is the angle that is either driven by a curve or inclination (yarn waviness or undulation), *h* is the change in height for a flow at a particular point, and *d* is the distance travelled by the flow. These latter length parameters vary with *x* and *z* in warp yarns, and *y* and *z* in weft yarns, respectively. The undulation angle θ changes along the yarn length, wherein the micro-flow tensor ($1/K_{t}$) is being evaluated in this regard. As it can be seen from Figure 3, the three yarn regions considered in the woven model include straight, curved and tangent segments, in which the two curves are connected with a tangent line. This connection between the two curved regions, is referred to as the tangent region. The UDF code employs a conditional statement to set criteria that could assign the appropriate equations to the right regions. The micro-flow is tracked with reference to *d* (yarn length), where the straight, curved and tangent segments are defined. The computational procedure is better captured by the flow Figure 4.

## 3. Results and Discussion

### 3.1. Mesh Dependency Study

**Figure 5:**
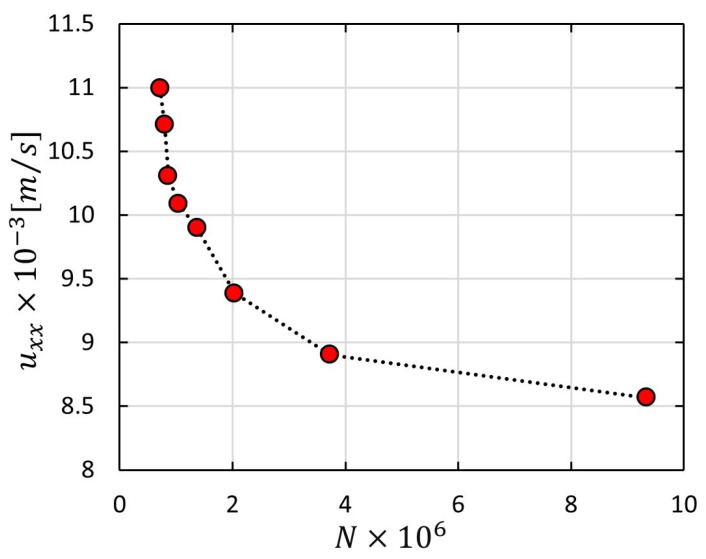
Mesh dependency study for channel flow case ($\phi_{s}=0.44$).

**Figure 6:**
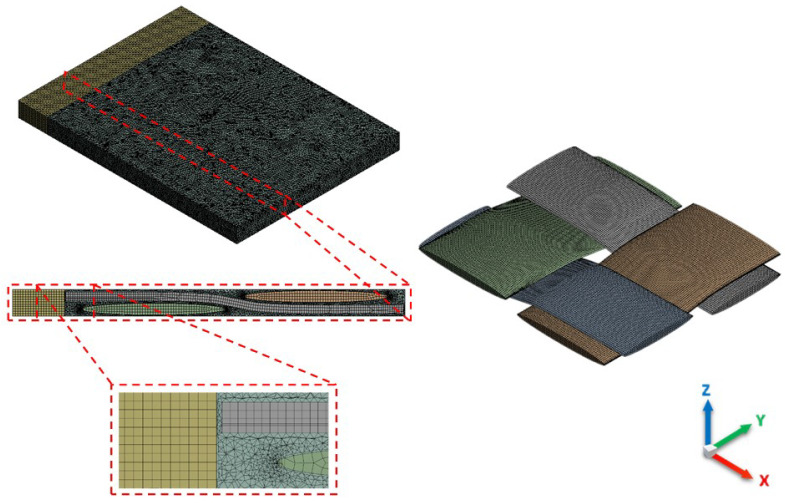
Multi-zone adaptive mesh (tetrahedrons/hexahedrons) for a woven model of elliptic tows ($\phi_{s}=44\%$).

All simulations are checked for mesh independency in order to obtain reliable results. Figure 5 represents a mesh dependency study for one of the simulations, where a sharp drop for velocity in *x*-direction is seen close to about 4 M mesh elements (*N*). At this point, the results tend to get more independent, wherein slight changes appear. In contrast to non-adaptive mesh, adaptive mesh offers better elements quality that exhibit proper alignment with boundaries as well as the possibility to reduce the number of cells, and hence it is used in this study to mesh local areas with a high degree of geometrical changes—see Figure 6. Geometrical details of warp and weft yarns are reported in Table 2, whereby a representative volume element (RVE) of a plain weave fabric is built as a rectangular domain 6.59 mm × 5.66 mm × 0.52 mm with an extra 1 mm inlet region. The width and spacing values of warp/weft yarns are varied in accordance with inter-tow porosities.

### 3.2. A Woven Fabric of Elliptic Permeable and Impermeable Tows

ANSYS Design Modeller allows the modelling of complex geometries with a variety of robust tools which derestrict the user to control such a model. It is equipped with multiple functions that enable creating, and modifying a geometry, from sketches to models, 2D or 3D, to a complete model for the analysis phase. It is worth mentioning that for more complex fibre/bundle topologies, other sophisticated open-sources such as TexGen can be used [44]. In this study, Design Modeler by ANYSYS is used to model textile structures and avoid any potential limitation in tools of other CAD software products.

Two design cases were selected based on the work conducted by Belov et al. [23] in regards to solid and porous tows of a woven model. Thereafter, a comparative analysis was performed to emphasise the reliability and applicability of the developed numerical tool and approach in this study for such a complex fabric architecture. The analysis characterises the influence of the intra-tow permeability on the overall permeability in a unit cell from solid (impermeable) to highly permeable tows along with different inter-tow porosities. This was done by modifying the cross-sectional area of the elliptical tows to obtain the low fibre volume fraction in a unit cell. The influence on the overall permeability $K_{xx}$ by the local permeability was checked versus three different sets of intra-tow permeabilities summarised in Table 3. The developed model followed steady, laminar, single-phase, and incompressible Newtonian fluid in which homogenous porous media and isotropic fabric were assumed throughout the numerical analysis. The flow density and viscosity were, respectively, ρ=1300kg/m${}^{3}$, μ=0.15Pa·s, with a pressure injection at 10kPa.

**Figure 7:**
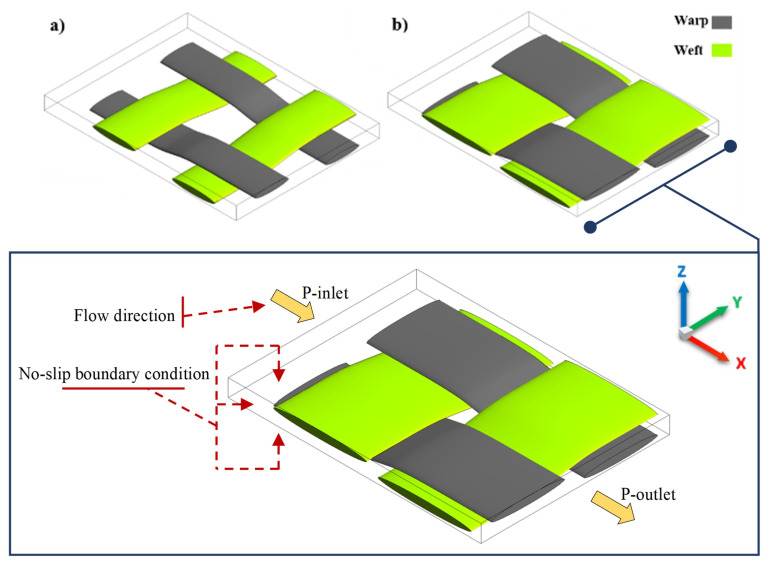
UC of 2D woven fabric with different inter-tow porosity of (**a**) $\phi_{s}=66\%$ (modified yarns cross-section 1.4 mm wide in warp and 1.8mm wide in weft direction, with a spacing of 1.39mm and 1.34mm, respectively), and (**b**) $\phi_{s}=44\%$ (geometrical details presented in Table 2).

Figure 7 depicts a woven fabric model with two different inter-tow porosities of 44% and 66%. The channel flow in the warp direction (*x*-coordinate) was considered for calculating the in-plane permeability. Wall boundary conditions were accounted for in the unit cell with no-slip at walls. The no-slip boundary condition is valid in a continuum/Darcy flow regime, hence for viscous fluids. This assumption allows easier implementation and computational efficiency in terms of meshing and flow calculations.

The ratio of the overall permeability $K_{o}$ to the inter-tow permeability $K_{s}$ was observed to be higher in the unit cell with $\phi_{s}=44\%$ in comparison to $\phi_{s}=66\%$. At $\phi_{s}=44\%$, $K_{o}/K_{s}$ depicts 1.72 for low permeable tows, while an increase was observed in high permeable tows, such as 2.17. In contrast, the unit cell with $\phi_{s}=66\%$ shows lower ratios of 1.29 and 1.42 for low and high permeable tows, respectively. The reason behind this is because the intra-tow flow at small cross-sectional yarns, which are lower in fibre volume fractions, has less of an impact on the inter-tow permeability. Whereas, at higher fibre volume fractions, as in the case of larger cross-sectional yarns, the intra-tow flow remarkably affects the inter-tow permeability, and hence the overall permeability.

Table 3 indicates a good agreement for the obtained results by the model developed in this study compared to those from Belov et al. [23] for both impermeable and permeable tows. The obtained numerical results for $K_{o}$ at low and high intra-tow permeability values, account for the local curvature (undulation) of the tows in which θ varied in the range of 0≤θ≤10.2 based on the woven fabric geometries, as detailed in Table 2. This highlights the reliability, capability and applicability of such a numerical tool to model and characterise resin impregnation in regular and complex fabric architectures during LCM. The slight discrepancies in results between the two studies would be attributable to that fact that a heterogenous porous medium was assumed by Belov et al. [23] and solved using LBM, which incorporates N–S and Brinkman equations for modelling flow inter-tow and intra-tow, respectively. Unlike this work, which assumes homogenous porous media wherein fibres/filaments within a fibre bundle are spaced and measured identically and have the same fabric properties.

Furthermore, this analysis is more customized in terms of the driven influence by local curvature within the woven yarns on resin flow. Since the available feature by ANSYS-fluent, which is the User-Defined Function (UDF), allows the user to customize the numerical model in respect of boundary conditions, material properties, source terms, etc. In contrast, the FlowTex tool, which was adopted by Belov et al. [23] based on LBM, constrained the user to the available functions, and that, for instance, did not account for the local curvature. This means ANSYS-Fluent can be confidently used and enhanced to solve the problem with more precision. It is reported by [23] that the LBM modelling has a time-consuming CPU; whereas, the adopted approach and modelling by this paper emphasises prompt and simple flow simulations, and subsequently, permeability predictions. The obtained numerical results show a good correlation with the work by Belov et al. [23], and imply 1–37% a mismatch error in comparison for a 2D woven UC.

**Figure 8:**
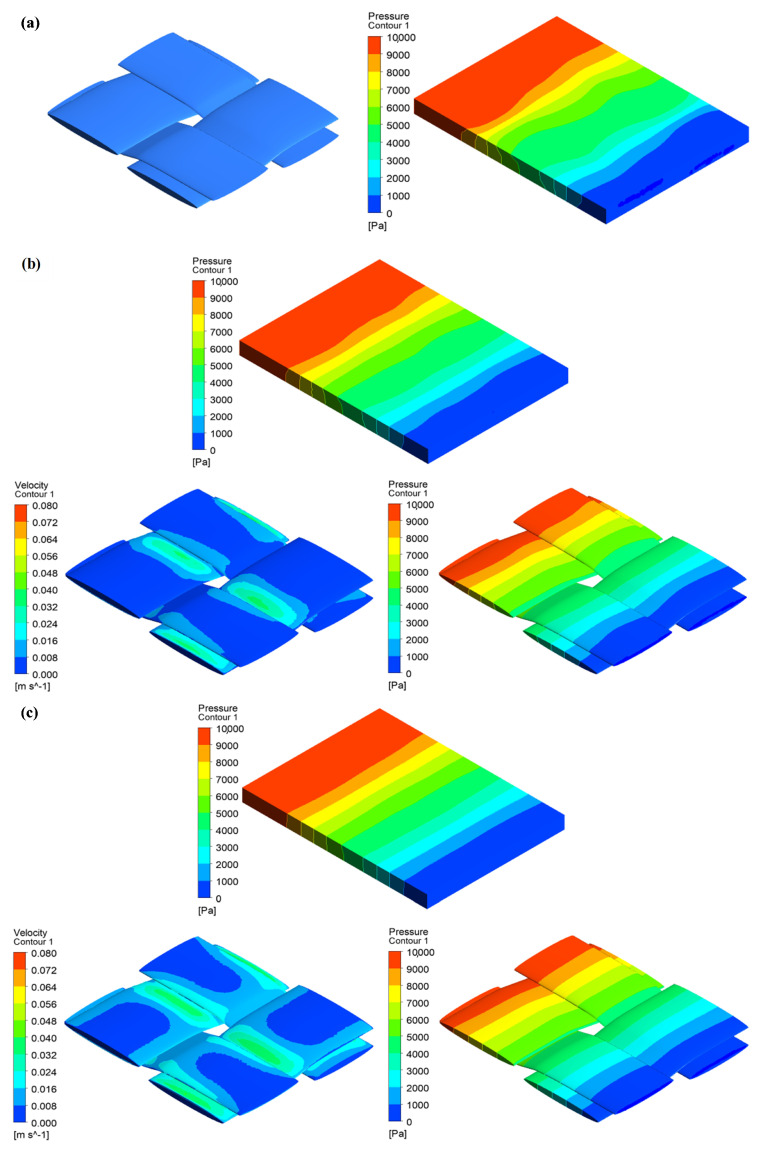
Pressure and velocity contours for channel flows in single- and dual-scale porous media for a unit cell with an inter-tow porosity value $\phi_{s}=44\%$ and local curvature of tows varied in the range of 0≤θ≤10.2: (**a**) impermeable tows, (**b**) permeable tows with $K_{t\|}=6.8\times 10^{-11}$, $K_{t⊥}=3.5\times 10^{-11}$ (**c**) permeable tows with $K_{t\|}=6.8\times 10^{-10}$, $K_{t⊥}=3.5\times 10^{-10}$.

Figure 8 shows pressure and velocity profiles for channel flows in single and dual-scale porous media for a unit cell with an inter-tow porosity ($\phi_{s}=44\%$). For permeable tows, pressure and velocity contours are also represented to mark change from the inlet to the outlet throughout the unit cell as well as the fibre bundles. As can be seen in Figure 8, namely part (b) and (c), velocity increase of the injected flow is exhibited in many locations throughout the fibre bundles, especially in (c), while they tend to be less, as shown in (b). This stems from the fact that intra-tow porosity plays a significant role for flow resistance to permeate inside the tows, which emphasizes the importance of dual-scale flow modelling when designing fibre-reinforced composites to assure good impregnation, thus high quality [16,45].

### 3.3. Degree of Local Curvature in Woven Tows

As mentioned earlier, a UDF was hooked into ANSYS-Fluent to simulate the flow inside woven tows that exhibit forms of curvature or tangent within a unit cell. This work performed a comparative analysis with and without applying the developed code, on various intra-tow porosity ($\phi_{t}$) values of 10%, 15%, 20%, 25%, 30%, 35%, 40%, and 45%. The fibre radius was kept constant ($R_{f}$ =10.5 μm) in Section 3.3 and Section 3.4, with the assumption of a parallel array of fibres (filaments) in a hexagonal packing arrangement. The unit cell domain was also unchanged, while the cross-sectional dimensions of tows were modified to 2.64mm wide (warp) and 3.12mm wide (weft direction), with spacing values of 0.32mm and 0.29mm, respectively. This modification gives rise to fibre volume fraction and brings greater binding of yarns, and, thereby, an increase in curvature. Longitudinal microscopic permeabilities in woven warp and weft yarns are subjected to the impact of a curvature degree or an angle by tangent; however, transverse microscopic permeabilities act perpendicularly to fibres or filaments throughout fibre bundles, hence they are not affected by the degree of local curvature. Since flow penetration into a homogenous isotropic porous medium is assumed, the in-plane permeabilities $K_{xx}$ and $K_{yy}$ are equal($K_{xx}=K_{yy}=K$), while the out-plane value ($K_{zz}$) is one to three orders of magnitude smaller. In such an isotropic case, the in-plane permeability becomes the permeability tensor or the overall permeability $K_{o}$ when ignoring the through-thickness permeability ($K_{zz}$), implying very small thickness in the mould cavity in comparison to mould width and length; however, this work considers the out-plane permeability and investigates its impact by the degree of local curvature on the longitudinal and transverse resin impregnations.

**Figure 9:**
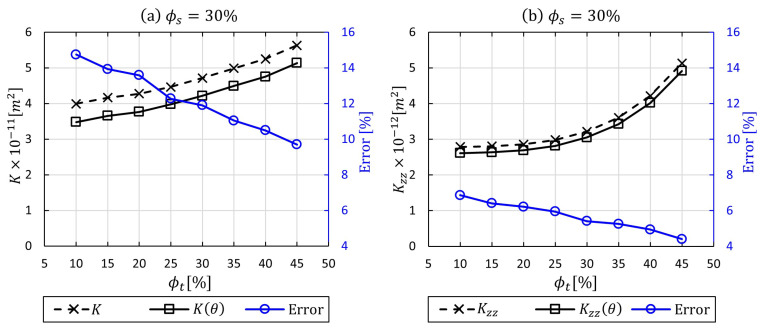
A comparative analysis of permeability with and without applying local curvature (0≤θ≤35) via UDF for permeable tows at inter-tow porosity ($\phi_{s}=30\%$) with various $\phi_{t}$ values: (**a**) in-plane permeability, (**b**) through-thickness permeability.

Figure 9 shows the obtained numerical results for *K* and K(θ) at low and high intra-tow porosity values, in which tow undulation (θ) varied in the range of 0≤θ≤35. The highest discrepancy in permeability was observed at $\phi_{t}=10\%$ with 14.75%, whereas the difference became less effective at high $\phi_{t}$ (relatively low fibre volume fraction). The variations appear to be insignificant for the out-plane permeability case ($K_{zz}$ and $K_{zz}\left(\theta \right)$), which could be explained by that fact that flow permeates across tows, hence penetrates perpendicular to fibres or filaments. The flow across tows will not be affected by local curvature, which is not the case for the flow along tows. The results highlight that for woven fabrics with low fibre volume fraction $V_{f}\leq 42\%$ ($\phi_{o}\geq 58\%$), there is an insignificant change in the predicted mesoscopic permeability when the local curvature of tows is incorporated. This can be observed at $\phi_{t}=45\%$ in Figure 9a, with <10% for the in-plane permeability. Nevertheless, this becomes more pronounced when $\phi_{t}<45\%$, with higher discrepancies, in which the fibre volume fraction increases beyond $V_{f}>42\%$ ($\phi_{o}<58\%$). This study suggests that in a low fibre volume fraction, the effect of yarn local curvature could be neglected; however, in cases of high fibre volume fraction, the curvature needs to be considered, since it affects the $K_{t}$ intra-tow permeability of the resin flow, which contributes to manufacturing quality and performance of the designed composite part.

### 3.4. Degree of Local Curvature in Multi-Layer Structure with Nesting

**Figure 10:**
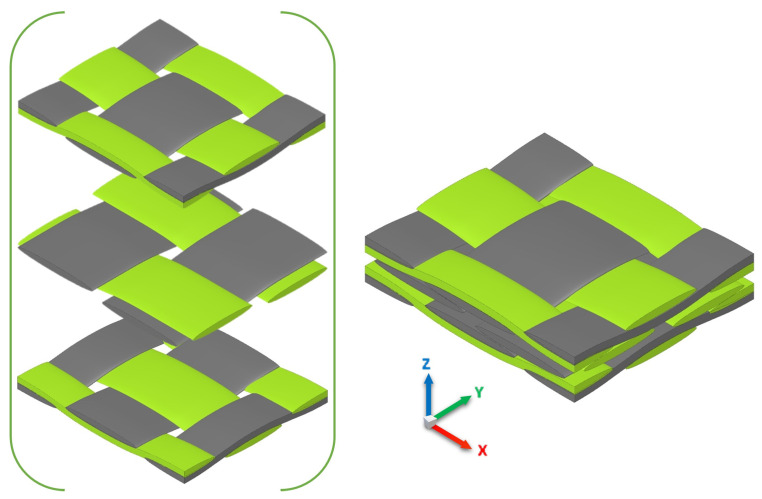
Stacking and nesting of layers in a laminate: three multi-layer woven model.

In real multi-layer fabrics, tows or yarns are subjected to compression during LCM processes such as the RTM filling process. Inter-tow and intra-tow porosities undergo changes that influence the thickness of tows as well as the gaps between fibre bundles or layer fabrics. This phenomenon is defined as nesting of layers that plays a significant role in the resin flow path, and hence dual-scale permeability variations. A considerable amount of research [25,46,47,48,49,50,51] has been conducted with regard to packing of multiple layers and nesting effects on permeability. Since the degree of local curvature appeared to influence the global permeability, as investigated in the previous section, it will be worthwhile to characterise resin flow in nesting multi-layer fabrics incorporating local tow curvature. Figure 10 represents the nesting model of a three-ply fabric unit cell. Unsheared or undeformed plain-weave fabric is considered in this study to achieve in-plane periodicity and simple reliable permeability approximation. The three-ply unit cell follows the detailed woven fabric geometries shown in Table 2; however, with nesting of layers that involves yarn surface contact and more binding. This would reduce the inter-tow porosity, but could increase yarn curvature. This unit cell has dimensions of a rectangular domain 6.59 mm × 5.66 mm × 1.385 mm with an extended inlet region 1 mm.

**Figure 11:**
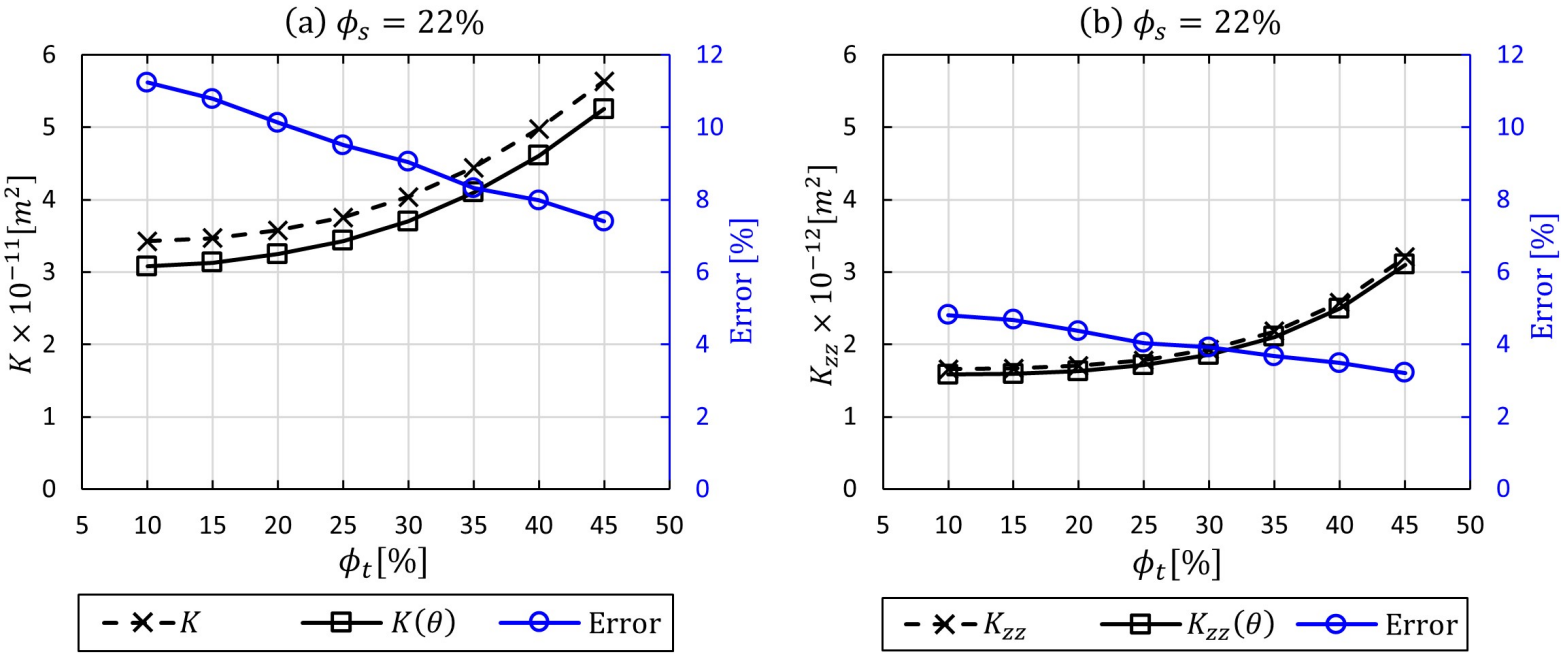
A comparative analysis of permeability with and without applying local curvature (0≤θ≤35) via UDF for flow throughout three-layer woven unit cell with nesting effect at inter-tow porosity ($\phi_{s}=22\%$) with various $\phi_{t}$ values: (**a**) in-plane permeability, (**b**) through-thickness permeability.

Figure 11 presents in-plane and through-thickness permeabilities calculated for a cell with three nesting layers of fabric in which the degree of local curvature is incorporated for three intra-tow porosities (10%, 15%, 20%, 25%, 30%, 35%, 40%, and 45%). Stacked woven layers reduce the inter-tow porosity in contrast to a single-layer woven model (no nesting) in the previous section, which stems from the fact that the number of gaps and spaces in-between yarns or tows are either being reduced or blocked. Thus, the global permeability is expected decrease. As can be seen in Figure 11, the local curvature impact still plays a substantial role in the nesting multi-layer woven model. The dual-scale in-plane permeability was affected by 11.23% in the high fibre bundle volume faction (90%), and the impact declined by 7.4% in the low fibre bundle volume factions 55%, respectively. In contrast, the dual-scale through-thickness permeability values show low differences ranging from 3.21% to 4.81%. In general, these lower impacts are attributable to the nesting effect, which contributes to surface contact of the fibre bundles to some extent. That would disturb the micro-flow within the yarns, and in this case, the local curvature concept would not be neccessary. This flow analysis allows the local curvature effect in the case of stacking and nesting of fabric layers to be taken into account.

## 4. Concluding Remarks

The present work developed a numerical model using ANSYS-Fluent for the prediction of dual-scale (micro- and meso-scale) permeability in woven composites fabricated by the resin transfer moulding (RTM) process. For a 2D woven fabric architecture, the study identified the influence of microscopic permeability on the overall permeability within a plain-woven unit cell model. The work was compared with the Lattice Boltzmann method (LBM) numerical results by Belov et al. [23]. The impact of the local permeability on the overall permeability appeared in low inter-tow porosity of the preform (≤44%), on the other hand, high inter-tow porosities (>44%) depicted the ineffectiveness of intra-tow flow on the overall flow [23]. A comparative analysis indicated a good agreement with those by LBM results.

A contribution to flow through porous media field was made by implementing an algorithmic code based on the degree of curvature (local tow/yarn undulation) and tangent equations to control longitudinal flows intra-tow. The results highlighted that for the structures with low fibre volume fraction, $V_{f}\leq 42\%$, there was a minor change in the predicted mesoscopic permeability when the local curvature of tows was incorporated into the numerical model. By contrast, the discrepancy was about 14% when the fibre volume fraction was increased. As this study suggested that in a low fibre volume fraction within the tows, the degree of local curvature could be neglected; however, in a high fibre volume fraction and higher curvature of woven fibre bundles, it is recommended to be followed, since it affects the resin flow, which might introduce defects that affect the performance of the designed component. 

## Figures and Tables

**Table 1:** Numerical modelling considering dual-scale porous media.

Reference	$K_{o}/K_{s}$	$\phi_{t}$ [%]	$\phi_{0}$ [%]	Packing Arrangement	Dual-Scale Approach
Sadiq et al. [22]	1.0406	25	61.6	Array of solid and porous	1D Darcy’s equation
	1.198	31	70.9	circular fibre bundles	
Ranganathan et al. [32]	1	30	45	Hexagonal arraignments	Stoke flow equations
					for open region and
	$K_{o}/K_{s}>1$	if $\phi_{t}>30$		in elliptic tows	Brinkman equation for
					porous region
Nedanov and Advani [33]	1.003	29	39	Hexagonal packing of	CFD package FIDAP,
					numerically solves
				fibres in woven fabric	stokes flow and
					Brinkman equation
Belov et al. [23]	1.25	42	66	A plain-woven fabric	Lattice Boltzmann
					method, WiseTex
					software
Tahir et al. [24]	1.03	25	62	Hexagonal arrangements of	ANSYS-Fluent, Navier-
				fibres in circular tows	stokes equations for
				within unit cell	dual scale
Syerko et al. [34]	4.6	36	62	Quadradic Packing	Applied Brinkman
					equations and mass
					conservation

**Table 2:** Details of the woven fabric geometries [23].

Parameters	Value	Units
Width warp yarns	2.21	mm
Gap warp yarns	0.58	mm
Width fill yarns	2.79	mm
Gap fill yarns	2.21	mm
Areal density	420	g/m${}^{2}$
Specific density	2520	kg/m${}^{3}$
Yarn tex warp	580	g/km
Yarn tex weft	600	g/km

**Table 3:** Summary of calculated global permeability in the *x*-direction ($K_{xx}$) using the developed numerical model in this study and their comparison with the results presented by Belov et al. [23]. Two different inter-tow porosities ($\phi_{s}$) were chosen with three different sets of intra-tow permeabilities ($K_{t\|}$ and $K_{t⊥}$).

$\phi_{s}\;$[%]	$K_{t}\;$[m${}^{2}$]	$K_{\mathrm{xx}}\;$[m${}^{2}$]	$K_{\mathrm{xx}}\;$[m${}^{2}$]	$K_{o}/K_{s}$
		Current Study	Belov et al. [23]	
44	$K_{t\|}=0$	$1.13\times 10^{-9}$	$1.55\times 10^{-9}$	1
	$K_{t⊥}=0$			
	$K_{t\|}=6.8\times 10^{-11}$	$1.93\times 10^{-9}$	$1.95\times 10^{-9}$	1.26−1.71
	$K_{t⊥}=3.5\times 10^{-11}$			
	$K_{t\|}=6.8\times 10^{-10}$	$2.45\times 10^{-9}$	$2.97\times 10^{-9}$	1.92−2.17
	$K_{t⊥}=3.5\times 10^{-10}$			
66	$K_{t\|}=0$	$3.48\times 10^{-9}$	$3.65\times 10^{-9}$	1
	$K_{t⊥}=0$			
	$K_{t\|}=6.8\times 10^{-11}$	$4.51\times 10^{-9}$	$4.70\times 10^{-9}$	1.29
	$K_{t⊥}=3.5\times 10^{-11}$			
	$K_{t\|}=6.8\times 10^{-10}$	$4.94\times 10^{-9}$	$5.65\times 10^{-9}$	1.42−1.55
	$K_{t⊥}=3.5\times 10^{-10}$			

## Data Availability

Data is contained within the article.

## Abbreviations

*Latin letters:*
*C*	Chord length, m
$D_{c}$	Degree of curvature
*d*	Distance, *m*
g	Gravitational acceleration, m/s${}^{2}$
*h*	Height of curvature, m
K	Permeability tensor, m${}^{2}$
*p*	Pressure, Pa
*R*	Radius, m
*S*	Source term
*t*	Time, s
u	Volume averaged velocity vector, m/s
$V_{f}$	Volume fraction
x,y,z	Global coordinate system
*Latin letters:*
θ	Rotation angle between global and principal coordinate system
μ	Dynamic viscosity, Pa·s
ρ	Density, kg/m${}^{3}$
τ	Stress tensor vector, Pa
ϕ	Porosity
*subscripts:*
*c*	Curvature
*f*	Fibre/filament
*o*	Overall/global
*t*	Intra-tow/microscopic/local/tow
*s*	Inter-tow/single scale
x,y,z	Global coordinate system
‖	Longitudinal/parallel
⊥	Transverse/perpendicular
*Abbreviation:*
CFD	Computational fluid dynamics
LBM	Lattice Boltzmann method
LCM	Liquid composite moulding
N-S	Naiver-Stokes
RTM	Resin transfer moulding
RVE	Representative volume element
UC	Unit cell
UDF	User-defined function

## References

[1] Gascón L., García J., LeBel F., Ruiz E., Trochu F. (2015). Numerical prediction of saturation in dual scale fibrous reinforcements during Liquid Composite Molding. Compos. Part A Appl. Sci. Manuf..

[2] Kuentzer N., Simacek P., Advani S.G., Walsh S. (2006). Permeability characterization of dual scale fibrous porous media. Compos. Part A Appl. Sci. Manuf..

[3] Tan H., Pillai K.M. (2012). Multiscale modeling of unsaturated flow in dual-scale fiber preforms of liquid composite molding I: Isothermal flows. Compos. Part A Appl. Sci. Manuf..

[4] Bodaghi M., Lomov S., Simacek P., Correia N., Advani S. (2019). On the variability of permeability induced by reinforcement distortions and dual scale flow in liquid composite moulding: A review. Compos. Part A Appl. Sci. Manuf..

[5] Liu B., Bickerton S., Advani S.G. (1996). Modelling and simulation of resin transfer moulding (RTM)—gate control, venting and dry spot prediction. Compos. Part A Appl. Sci. Manuf..

[6] Li S., Gauvin R. (1991). Numerical analysis of the resin flow in resin transfer molding. J. Reinf. Plast. Compos..

[7] Bruschke M., Advani S. (1994). A numerical approach to model non-isothermal viscous flow through fibrous media with free surfaces. Int. J. Numer. Methods Fluids.

[8] Phelan F.R. (1997). Simulation of the injection process in resin transfer molding. Polym. Compos..

[9] Soukane S., Trochu F. (2006). Application of the level set method to the simulation of resin transfer molding. Compos. Sci. Technol..

[10] Parnas R.S., Phelan F. (1991). The effect of heterogeneous porous media on mold filling in resin transfer molding. Sampe Quart.

[11] Binetruy C., Hilaire B., Pabiot J. (1997). The interactions between flows occurring inside and outside fabric tows during RTM. Compos. Sci. Technol..

[12] Lekakou C., Bader M. (1998). Mathematical modelling of macro-and micro-infiltration in resin transfer moulding (RTM). Compos. Part A Appl. Sci. Manuf..

[13] De Parseval Y., Pillai K., Advani S.G. (1997). A simple model for the variation of permeability due to partial saturation in dual scale porous media. Transport Porous Med..

[14] Pillai K., Advani S. (1998). A model for unsaturated flow in woven fiber preforms during mold filling in resin transfer molding. J. Compos. Mater..

[15] Wang Y., Grove S. (2008). Modelling microscopic flow in woven fabric reinforcements and its application in dual-scale resin infusion modelling. Compos. Part A Appl. Sci. Manuf..

[16] Gascón L., García J., LeBel F., Ruiz E., Trochu F. (2016). A two-phase flow model to simulate mold filling and saturation in Resin Transfer Molding. Int. J. Mater. Form..

[17] Parnas R.S., Howard J.G., Luce T.L., Advani S.G. (1995). Permeability characterization. Part 1: A proposed standard reference fabric for permeability. Polym. Compos..

[18] Zhong W., Xing M., Chen S. (2004). A statistic model of mold filling through fibrous structures. J. Compos. Mater..

[19] Wu C.H., James Wang T., James Lee L. (1994). Trans-plane fluid permeability measurement and its applications in liquid composite molding. Polym. Compos..

[20] Spaid M.A., Phelan F.R. (1998). Modeling void formation dynamics in fibrous porous media with the lattice Boltzmann method. Compos. Part A Appl. Sci. Manuf..

[21] Verrey J., Michaud V., Månson J.A. (2006). Dynamic capillary effects in liquid composite moulding with non-crimp fabrics. Compos. Part A Appl. Sci. Manuf..

[22] Sadiq T., Advani S., Parnas R. (1995). Experimental investigation of transverse flow through aligned cylinders. Int. J. Multiph. Flow.

[23] Belov E.B., Lomov S.V., Verpoest I., Peters T., Roose D., Parnas R., Hoes K., Sol H. (2004). Modelling of permeability of textile reinforcements: Lattice Boltzmann method. Compos. Sci. Technol..

[24] Tahir M.W., Hallström S., Åkermo M. (2014). Effect of dual scale porosity on the overall permeability of fibrous structures. Compos. Sci. Technol..

[25] Senoguz M., Dungan F., Sastry A., Klamo J. (2001). Simulations and experiments on low-pressure permeation of fabrics: Part II—The variable gap model and prediction of permeability. J. Compos. Mater..

[26] Weitzenböck J., Shenoi R., Wilson P. (1999). Radial flow permeability measurement. Part A: Theory. Compos. Part A Appl. Sci. Manuf..

[27] Chan A.W., Hwang S.T. (1991). Anisotropic in-plane permeability of fabric media. Polym. Eng. Sci..

[28] Weitzenbock J.R. (1996). Flow Characterization in Resin Transfer Moulding. Ph.D. Thesis.

[29] Parnas R.S., Salem A.J., Sadiq T.A., Wang H.P., Advani S.G. (1994). The interaction between micro-and macro-scopic flow in RTM preforms. Compos. Struct..

[30] Chan A.W., Larive D.E., Morgan R.J. (1993). Anisotropic permeability of fiber preforms: Constant flow rate measurement. J. Compos. Mater..

[31] Lei G., Dong P., Mo S., Yang S., Wu Z., Gai S. (2015). Calculation of full permeability tensor for fractured anisotropic media. J. Pet. Explor. Prod. Technol..

[32] Ranganathan S., Phelan F.R., Advani S.G. (1996). A generalized model for the transverse fluid permeability in unidirectional fibrous media. Polym. Compos..

[33] Nedanov P.B., Advani S.G. (2002). Numerical computation of the fiber preform permeability tensor by the homogenization method. Polym. Compos..

[34] Syerko E., Binetruy C., Comas-Cardona S., Leygue A. (2017). A numerical approach to design dual-scale porosity composite reinforcements with enhanced permeability. Mater. Des..

[35] Gebart B.R. (1992). Permeability of unidirectional reinforcements for RTM. J. Compos. Mater..

[36] Skartsis L., Khomami B., Kardos J. (1994). A semi-analytical one-dimensional model for viscoelastic impregnation of fibrous media. Adv. Mater..

[37] Westhuizen J., Plessis J.P.D. (1994). Quantification of unidirectional fiber bed permeability. J. Compos. Mater..

[38] Neuman S.P. (1977). Theoretical derivation of Darcy’s law. Acta Mech..

[39] Luminari N. (2018). Modeling and Simulation of Flows over and through Fibrous Porous Media. Ph.D. Thesis.

[40] Whitaker S. (1986). Flow in porous media I: A theoretical derivation of Darcy’s law. Transp. Porous Med..

[41] Zijl W., Stam J. (1992). Modeling permeability in imperfectly layered porous media. I. Derivation of block-scale permeability tensor for thin grid-blocks. Math. Geol..

[42] Hoes K., Dinescu D., Sol H., Vanheule M., Parnas R.S., Luo Y., Verpoest I. (2002). New set-up for measurement of permeability properties of fibrous reinforcements for RTM. Compos. Part A Appl. Sci. Manuf..

[43] Luo Y., Verpoest I., Hoes K., Vanheule M., Sol H., Cardon A. (2001). Permeability measurement of textile reinforcements with several test fluids. Compos. Part A Appl. Sci. Manuf..

[44] Long A., Brown L. (2011). Modelling the geometry of textile reinforcements for composites: TexGen. Composite Reinforcements for Optimum Performance.

[45] Hwang W.R., Advani S.G. (2010). Numerical simulations of Stokes–Brinkman equations for permeability prediction of dual scale fibrous porous media. Phys. Fluids.

[46] Šimáček P., Advani S.G. (1996). Permeability model for a woven fabric. Polym. Compos..

[47] Chen B., Chou T.W. (2000). Compaction of woven-fabric preforms: Nesting and multi-layer deformation. Compos. Sci. Technol..

[48] Dungan F., Senoguz M., Sastry A., Faillaci D. (2001). Simulations and experiments on low-pressure permeation of fabrics: Part I—3D modeling of unbalanced fabric. J. Compos. Mater..

[49] Saunders R., Lekakou C., Bader M. (1998). Compression and microstructure of fibre plain woven cloths in the processing of polymer composites. Compos. Part A Appl. Sci. Manuf..

[50] Lomov S.V., Verpoest I., Peeters T., Roose D., Zako M. (2003). Nesting in textile laminates: Geometrical modelling of the laminate. Compos. Sci. Technol..

[51] Grujicic M., Chittajallu K., Walsh S. (2004). Effect of shear, compaction and nesting on permeability of the orthogonal plain-weave fabric preforms. Mater. Chem. Phys..

